# Silver Sulfadiazine's Effect on Keratin-19 Expression as Stem Cell Marker in Burn Wound Healing

**DOI:** 10.37796/2211-8039.1014

**Published:** 2020-06-05

**Authors:** Dewi Sukmawati, Astheria Eryani, Lia Damayanti

**Affiliations:** 1 Department of Histology, Faculty of Medicine Universitas Indonesia, Jln. Salemba Raya No. 6 Jakarta, 10430, Jakarta, Indonesia; 2 Department of Histology, Faculty of Medicine Tarumanagara University, Jln. Letjen S. Parman No.1, Tomang, Grogol Petamburan, Jakarta, 11440, Indonesia

**Keywords:** silver sulfadiazine, keratin-19, epidermal stem cell, burn wound healing

## Abstract

**Background:**

Burn wounds are one of the causes of cutaneous injury that involve both epidermal and dermal layers of skin. Silver sulfadiazine (SSD) has been widely used to treat burn wounds, however recent studies have found the treatment to have some drawbacks, such as cellular toxicity effects. Cutaneous wound regeneration is known to start from the basal layer of the epidermal epithelial cells, which are enriched with highly proliferative cells. Keratin-19 (K19) is one of the epidermal stem cell biomarkers found in the skin. This study aims to explore the expression of K19 in burn wound tissue and to investigate the effect of SSD on its expression.

**Methods:**

We created a burn wound model in Sprague Dawley rats and randomly divided them into control and SSD groups. Wound closure was evaluated (visitrak) overtime series followed by histological evaluation of K19 expression in the wound tissue (immunohistochemistry staining).

**Results:**

Our model successfully represents full-thickness damage caused by a burn wound. The SSD group showed a faster reduction of wound surface area (wound closure) compared to the control group with the peak at day 18 post wounding (*p* < 0.05). K19 expression was found in both groups and was distributed on epidermal layers, hair follicles and dermis of granulation tissue showing similar patterns.

**Conclusion:**

Topical application of SSD on burn wounds showed superiority in wound closure and is likely to have no harmful effect on epidermal stem cells. However, further study is required to investigate the effect of silver species on cell viability and toxicity effects during long term treatment.

## 1. Introduction

Burn wounds can be devastating due to their longer healing period, which is often followed by incomplete healing with scar formation and functional or esthetic impairment [1–3]. The most common treatment for burn wounds is silver sulfadiazine (SSD), which has been used for decades for its antimicrobial activity. The active component of silver is in ionic form (Ag^+^), which commonly known as a safe, broad-spectrum antimicrobial agent. The availability of silver ions is dependent upon dissociation of silver salts or on their solubility in wound fluids, such as wound exudate. This ionic silver will interact with functional organic groups such as thiols, which act as the key components of prokaryotic cell wall structures and nucleic acids (DNA) which results in membrane porations and causes cell damage or a reduced ability to proliferate [4–7]. This interaction is the basic antimicrobial mechanism of SSD.

Prior clinical evidence of additional beneficial effects of the antimicrobial mechanism of SSD on wounds was shown by Geronemus et al. (1979), which showed that there was increased epithelialization of clean wounds in pigs of up to 28% associated with topical use of SSD (Silvadene ™) [4]. The results of this study were confirmed in further studies treating infection in acute [8] and chronic wounds [9]. Additional benefits of SSD on wound healing are improved rates of complete wound healing [10], effective wound cleansing and increased formation of granulation tissue [4], promotes early phase of wound healing, and increased local zinc and calcium concentrations, which further promotes epithelialization [11]. Moreover, 1% SSD (Flammazine®) has shown to be effective and safe for the treatment of wound infections in children 2 to <18 years [12].

Despite its advantages, some adverse effects have also been reported associated with the treatment of wounds with SSD and products containing it. SSD is used to prevent wound colonization and resulting infection by pathogenic bacteria. However, local toxicity on some cells has also been described. In vitro studies have been performed in fibroblasts, keratinocytes, and other human cell lines [13–16]. In one study, the application of SSD 100 μg/mL to culture media was demonstrated to cause morphological changes in fibroblasts during cell culture [14]. Another study showed a toxic effect of SSD in monolayer fibroblast cell cultures at a dose of more than 3.7×10 4%, while a dose of 50 × 10–4% was lethal for keratinocytes [16]. The cytotoxicity of silver-based dressings was also described and is associated with the release of silver from the dressings, as measured by silver concentration in the culture medium [13]. This cytotoxicity effect was proposed as the underlying mechanism for delays in wound closure and complete healing, as previously described [17–21].

Thus, although SSD is part of the standard treatment modality for burns, there is still conflicting data on whether SSD promotes or retards wound healing. Wound healing is a complex mechanism, especially in burn wounds. Burn injury represents cellular stress in the skin and carries the risk of infection and hypertrophic scars. The healing process in burns may consist of an inflammatory phase, proliferative phase, and remodeling phase. The last two phases are known to involve resident cells residing within skin layers and the skin's appendages [22–25].

The dynamic turnover of the epithelial cells plays an important role in maintaining the continual regeneration of skin throughout life both normally and in the injured condition. This process is performed by the population of stem cells resident within the skin. These cells are known to reside in the epidermal layer, follicular layer, sebaceous glands, and dermis layer of the skin [26–30]. Keratin 19 (K19) has recently been proposed as one of the stem cell biomarkers in cutaneous tissue [31–34]. Despite the cytotoxicity of SSD on some cells, less is known about the effect of topical SSD on epidermal stem cells in an injury setting. In this study, we aim to investigate the effect of topical SSD on burn wound healing and its effect on the expression of K19 in wound tissue.

## 2. Materials and Methods

### 2.1. Wound creation and animal groups

The wounds were created as the previous study done by our group [35,36]. We used a control group (C), which had burn wounds not treated with any intervention (only normal saline was applied); and a SSD group, which received once-daily topical SSD (Burnazine, Darya Varia) as a treatment for the wound. Wound closure was measured using visitrak as previously described, and the wound surface area was calculated in cm^2^ at days 0, 3, 6, 12, 18, and 24 post-wounding [35]. All the experiments in this study were approved by the Ethical Committee Faculty of Medicine Universitas Indonesia (Ethical approval No. 494/UN2.F1/ETIK/2016).

### 2.2. Burn wound tissue samples and histological routine staining

The wound tissues were collected on days 7, 14, and 21 post-wounding. All samples were then processed for histological slides with 5 μm thickness. For histological basic morphology, paraffin-embedded sections were stained with routine hematoxylin and eosin (H&E) as previously described [35].

### 2.3. Keratin-19 immunohistochemistry

The sections were deparaffinized in xylol and rehydrated. After a tris-buffered saline (TBS) wash, endogenous peroxidase was blocked by 3% hydro-peroxide (H2O2) for 15 minutes in the moisture chamber. After the wash with TBS, the sections were embedded in buffer citrate and put in a de-cloaking chamber at a temperature of 90 °C for 45 minutes for antigen retrieval. After a wash with TBS, the sections were incubated with 100 μL anti-CK19 mouse monoclonal antibody 1:300 dilutions (Biocare medical, CM 242A) and incubated at 4 °C overnight. The next day, after washing with TBS, the sections were incubated in Histofine Simple Stain Rat.

MAX PO (Nichirei Biosciences Inc.) at room temperature for 40 minutes. This reagent is composed of antibody conjugated polymers with peroxidase. After the TBS wash, the sections were incubated with aminoethyl carbazole (AEC) solution (Histofine Simple Stain AEC Solution; peroxidase chromogen/substrate solution; Nichirei Biosciences Inc.) for 10 minutes at room temperature and counterstained with hematoxylin. The K19 expression was quantified using an intensity score according to the scale: 0 = negative, +1 = weak but significant positivity, +2 = medium positivity and +3 = strong positivity, as previously described [37,38].

### 2.4. Data analysis

The numerical data of the wound area was analyzed with Student t-test and presented as mean ± SEM. Statistically significant was considered for the analysis results of *a p*-value less than 0.05. The histological slides were analyzed descriptively to explore the expression of K19 protein and its distribution on wound tissue; the quantification of K19 expression was using median data.

## 3. Results

### 3.1. Basic wound morphology

**Fig. 1:**
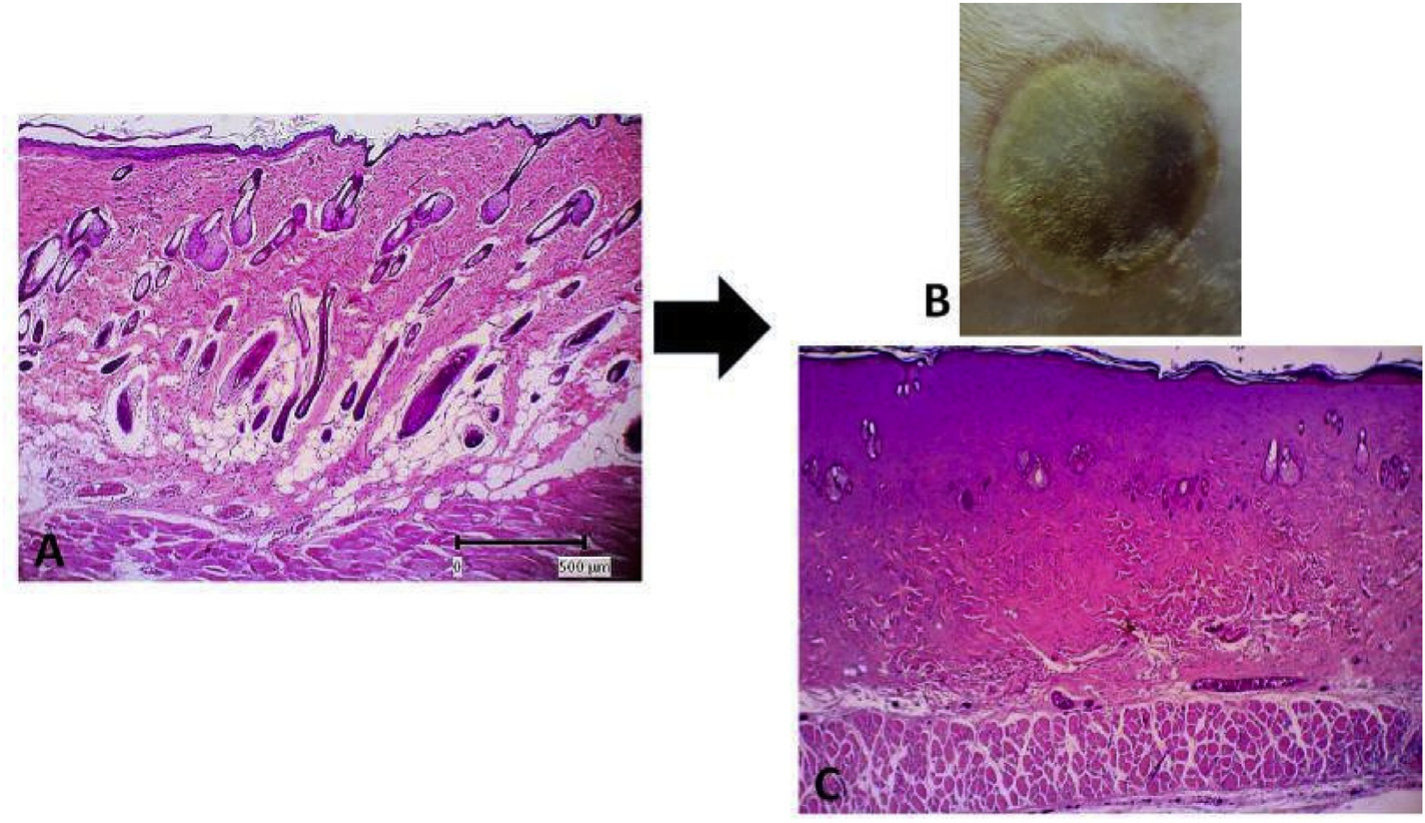
Thermal wound creation. A. Normal skin B. Wound (area) appearance on day 3 post wounded C. histological features of the wound on day 3 post wounded.

Using the method of wound creation previously used by our group, we confirmed that the burn wound caused full-thickness damage as shown in Fig. 1 (H&E staining). In this model, the pre-heated metal plate had successfully induced denaturation damage in the epidermal and dermal layers of the skin; therefore, up to day 3 post-wounding, it is difficult to observe the detail part of the skin compared to normal skin structure.

### 3.2. Wound closure

**Fig. 2:**
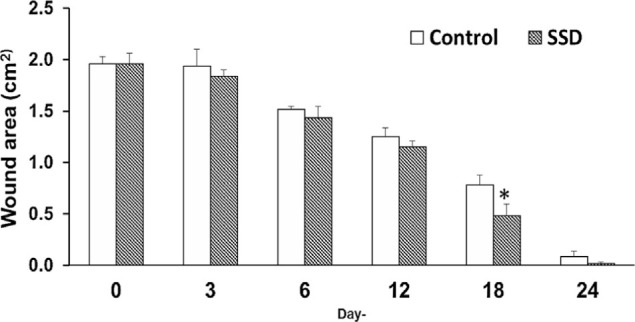
Measurement of wound size in time courses, presented as wound area in. cm^2^. SSD = silver sulfadiazine, *p < 0.05.

The surface area of the wound was observed and the wound area calculated; reductions in wound surface area were taken to represent wound closure and healing. Our results showed that the SSD group presented smaller wound surface areas (increased wound closure) than control group starting at day 6 and reaching a peak at day 18 (*p* < 0.05), then completely closing at day 24 post-wounding (Fig. 2). Therefore, the SSD group demonstrated faster-wound closure in full-thickness damage from burn wounds compared to the control group.

### 3.3. K19 expression in burn wound tissue

**Fig. 3:**
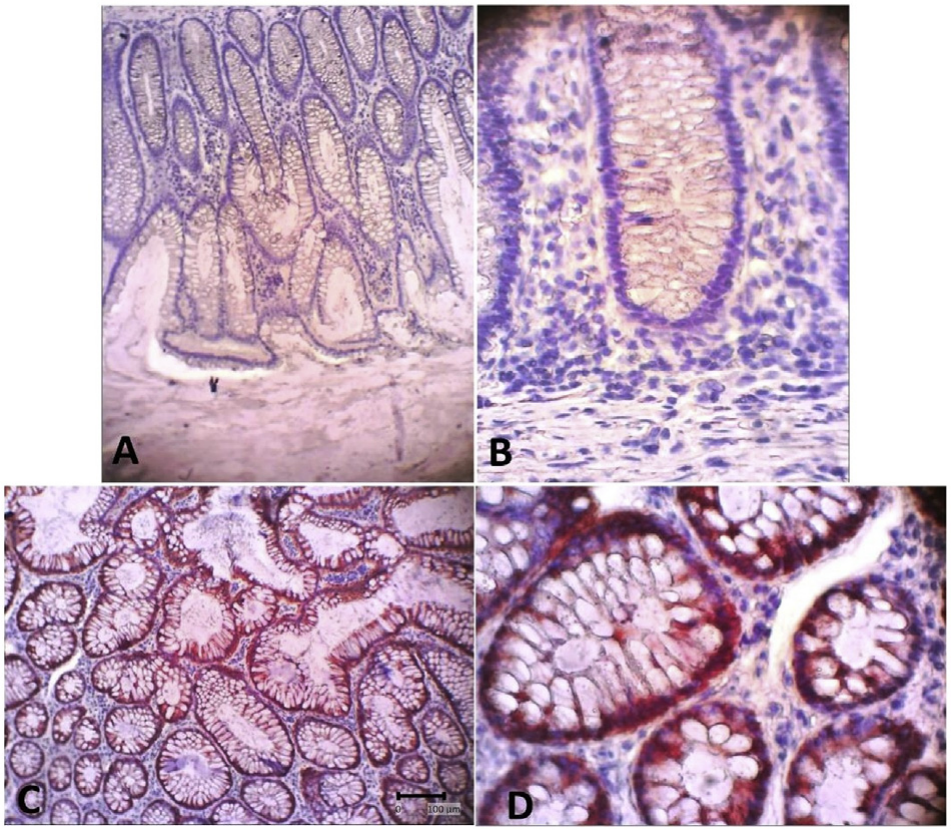
Positive Keratin-19 expression in human colon cancer tissue as a positive control. A&C = 1O×, B&D = 40× of magnification.

**Fig. 4:**
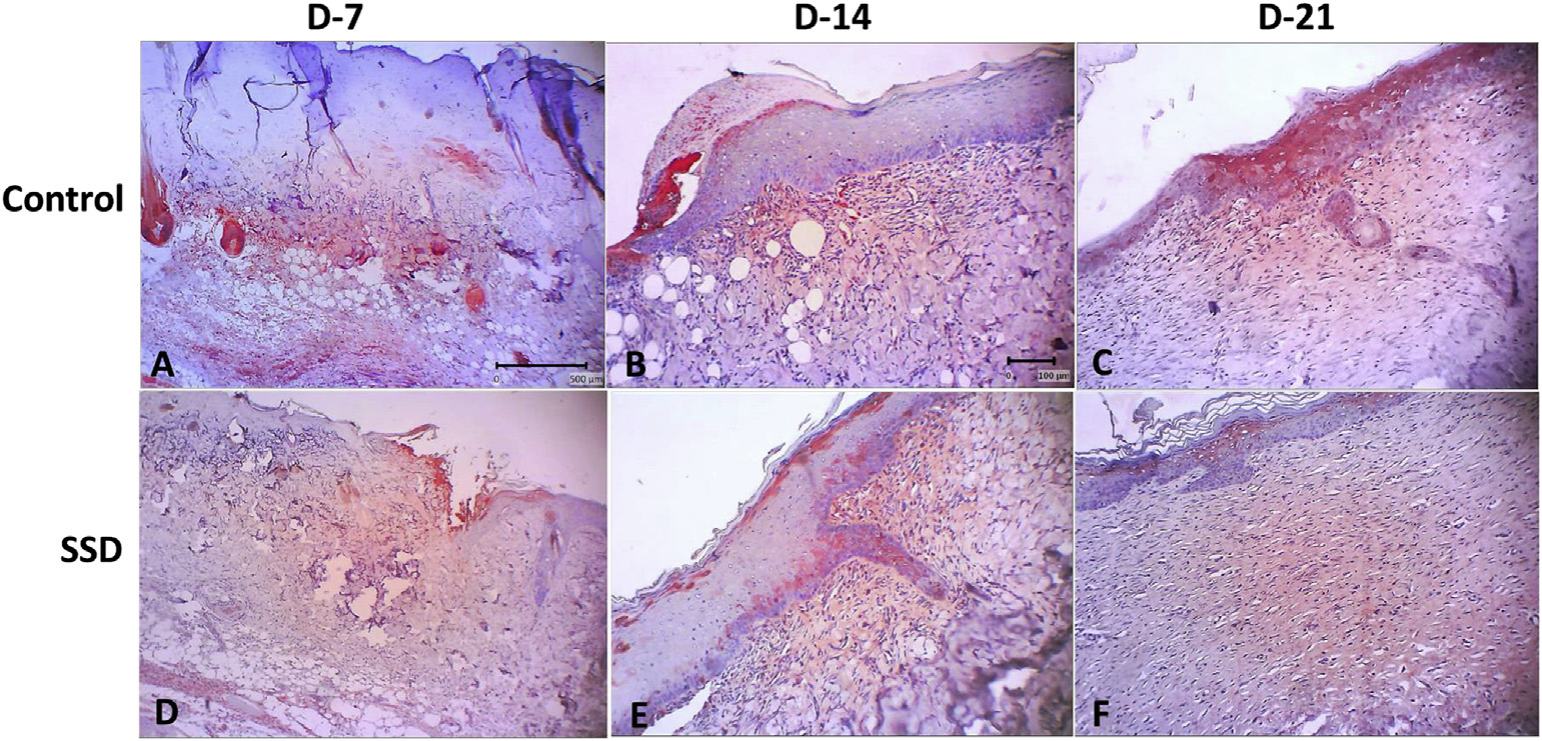
Keratin-19 expression in time course during burn wound healing. process. A&D = 4×, B–C and E-F = 10× of magnification.

**Fig. 5:**
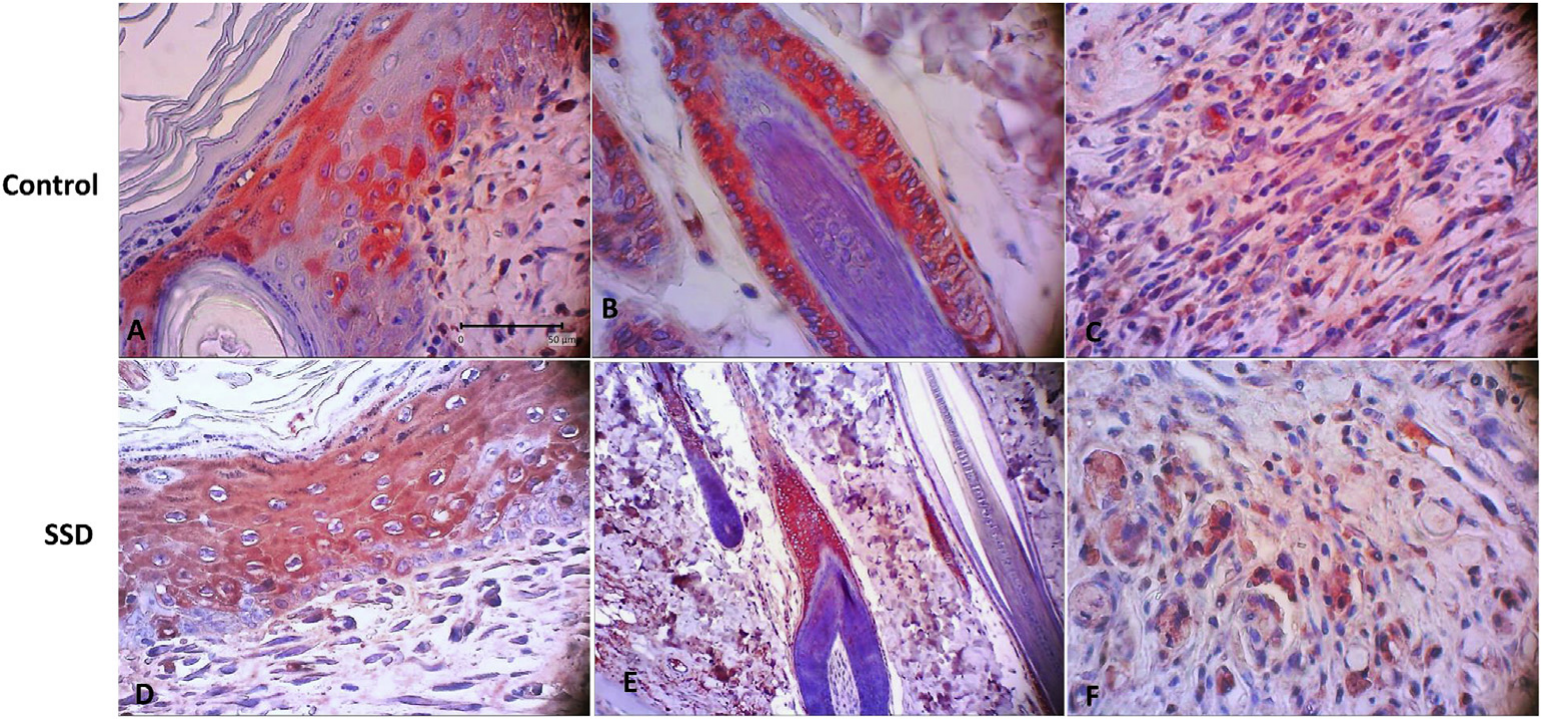
Distribution of Keratin-19 expression during wound healing process. Keratin- 19 is positively expressed in both groups which distributed on epithelial cells, hair follicle and fibroblast (red color).

**Fig. 6:**
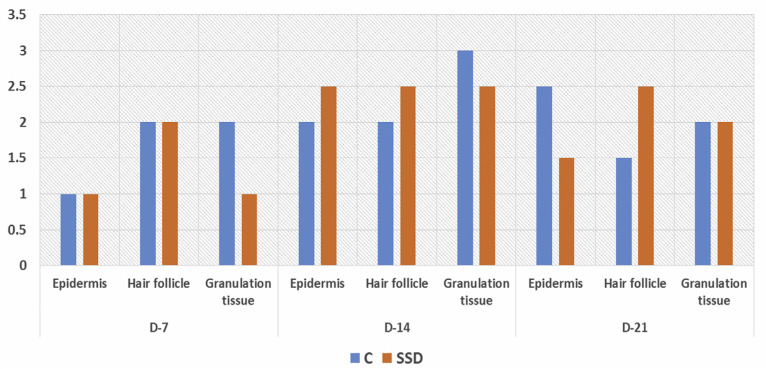
Expression intensity and distribution of Keratin-19 during wound healing process. Keratin-19 is positively expressed on epidermis layer, hair follicle and granulation tissue. Both group constantly expressed K-19 which distributed according to where stem cell were discovered in other studies including epidermis layer, hair follicle and granulation tissue. Notes: 0 = negative, +1 = weak but significant positivity, +2 = moderate positivity and +3 = strong positivity.

For immunohistochemistry staining, we used mouse anti-CK-19 monoclonal antibodies with colon cancer tissue as the positive control (Fig. 3). Immunohistochemical analysis of K19 expression, a marker of the presence of epidermal stem cells, showed that both groups expressed K19 during the wound healing process on days 7, 14, and 21 post-wounding (Fig. 4). K19 expression was evident in all layers of the skin and its appendages. In the epidermis layer, K19 was expressed in almost all layers. In the hair follicle, the K19 was expressed mainly in the outer root sheath. K19 was also expressed in the granulation tissue of the dermis layer (Fig. 5). Both the control and treatment groups showed similar patterns of K-19 distribution. Quantification of the intensity of K19 expression in burn wound tissues varied by location and time of observation. On day 7 post-wounding, both groups showed a weak expression of K19 within the epidermal layer, while the intensity of expression in the hair follicle was moderate. On day14 both groups showed a higher intensity of K19 expression in all locations than on day 7. In the SSD group, the intensity of K19 expression in the hair follicle remained high from day 7 to day 21, while the intensity of expression in the epidermis layer decreased. In the control group, the intensity of expression in the epidermis layer increased between day 7 and day 21, while expression in the hair follicle decreased (Fig. 6).

## 4. Discussion

Burn wounds are unique because of the effect of heat injury on the cutaneous tissue, which damages cells, connective tissue, and vascular components. In this study, our burn model successfully created full-thickness skin damage. The pre-heated metal used in this study positively damaged the tissue components and caused coagulation in the epidermis and dermis, followed by collagen denaturation and further necrosis, as shown in Fig. 1. This result was in line with several studies that previously described this method [39,40], and thus our model can properly facilitate the aim of this study.

SSD is widely used as one of the standard treatments for burns. Its primary mechanism is as a topical antimicrobial [11,41–43]. By controlling the bacteria growth within the burns wound environment, SSD is shown to also control wound healing [11,42]. Our study demonstrated that SSD positively promoted wound closure, which averaged faster than in the control group up to day 12 post-wounding. This effect peaked on day 18 when the treatment group showed significantly smaller wound areas and continued until the treatment group wounds completely closed on day 24. Despite the controversy over the effect of SSD on wound healing, our results support the use of SSD in promoting wound healing in line with previous studies [5,11,44,45]. Differing results found in other studies may be attributable to the use of different species (animal vs human) in research as well as the depth of the wound, which affects the healing time.

Despite its benefit as a topical antimicrobial, several studies have reported a cytotoxicity effect of SSD on some cells [13–16,46]. However, the effect of SSD on epidermal stem cells has not yet been reported. The dynamic of cellular homeostasis in the skin during physiological cell sloughing and regeneration is maintained by several stem cell populations residing within the skin layers and skin appendages [31-33,47–49]. In this study, to our knowledge, we are the first to report the effect of topical SSD on K19 expression, which is a known epidermal stem cell marker, in burn wound tissue. Our results demonstrated that K19 was expressed in both the SSD and control groups. These K19 expressions were distributed throughout the epidermal layer, in the outer root sheath of the hair follicle, and in granulation tissue within the dermis layer. These locations matched those described in previous studies [28,29,31,50,51]. In addition, the intensity of K19 expressions in the SSD group showed a similar pattern as in control group: weak-to-moderate intensity by 7 days post-wounding, a gradual increase to moderate-to-strong intensity by day 14, and a return to moderate-to-weak intensity by 21 days post-wounding. These patterns are in line with the physiology cutaneous healing process, which mainly active on days 7 –14 due to re-epithelialization and formation of the granulation tissue, which were then gradually decreased upon day-21 post wounded [2,25,40,52–54].

Our results showed that topical SSD for burn wounds is beneficial to burn wound healing. Moreover, the application of SSD up to 21 days post-wounding did not demonstrate toxicity to epidermal stem cells as shown by the similar expression patterns of K19 in the SSD group and the control group. Further study is required to confirm the possible direct cytotoxicity of SSD on epithelial stem cells under in vitro study. The intensity of K19 expression was based on a semi-quantitative score, and therefore further study is required to quantitatively quantify the value of the expression.

## 5. Conclusion

Our results show that SSD has a positive effect on burn wound healing, promoting faster wound closure compared to the control group. Moreover, our results showed for the first time the nontoxic effect of SSD on epidermal stem cells, indicated by levels of K19. Further study is required to determine the effect of SSD on living epidermal stem cells and quantify their viability after SSD exposure.
